# Introduction to Special Issue on Metabolic Imaging and Spectroscopy, 2025

**DOI:** 10.1117/1.JBO.30.S2.S23900

**Published:** 2025-12-11

**Authors:** Lin Z. Li, Arjun G. Yodh

**Affiliations:** University of Pennsylvania, Philadelphia, Pennsylvania, United States

## Abstract

The editorial provides an introduction for the JBO Special Issue on Metabolic Imaging and Spectroscopy.

The pathologies of many diseases—including cancer, diabetes, and cardiovascular, lung, skeletomuscular, neurological, and neurodegenerative diseases—are known to exhibit metabolic abnormalities with associated spatial heterogeneities. Indeed, metabolism-based imaging and spectroscopy of tissue offers relatively untapped contrasts and biomarkers for clinical diagnosis and treatment of disease. Traditional clinical biopsy for these pathologies, however, is invasive and suffers from severe under-sampling. As a result, over the last 50 years, we have witnessed increased interest in and demand for noninvasive or minimally invasive methods to probe tissue metabolism. Notably, over this same period, the field of metabolic imaging and spectroscopy has flourished, as evidenced by the number of new technologies developed for this purpose and by an upsurge in publications on the subject.

This special issue spans the field of metabolic imaging and spectroscopy. It was organized, in part, as a follow-up to the Third Britton Chance International Symposium on Metabolic Imaging and Spectroscopy, which was held at the University of Pennsylvania in 2024 (July 17–20). The symposium marked the 110th Birthday of Britton Chance, thereby honoring a founding father of metabolic imaging and spectroscopy. The symposium brought together physicists, engineers, biologists, and clinicians for presentation and discussion of cutting-edge research innovation and clinical translation in the field. The contributions were derived from many technologies, including optics, nuclear imaging (e.g., PET), NMR, EPR, ultrasound, and EEG. Approximately 165 participants from North America, Europe, and Asia attended the conference, which featured 66 oral presentations and 34 posters. The symposium triggered the submission of about 25 manuscripts to associated peer-reviewed journal issues on metabolic imaging and spectroscopy in the *Journal of Biomedical Optics (JBO)* or *Academic Radiology*, depending on topical focus.

The *JBO* issue focuses exclusively on optics. It comprises 15 manuscripts, including four review papers and eleven research papers. The four review papers offer snapshots of different subfields of metabolic imaging. A major focus concerns study of mitochondrial metabolism based on intrinsic fluorescence of NADH and oxidized flavoproteins containing FAD, a subject founded by Chance and collaborators in the 1950-60s. Mayevsky^1^ reviews the historic development (since the 1970s) of fiberoptic fluorometry for measuring brain mitochondrial NADH redox state *in vivo*. Georgakoudi et al.^2^ present a consensus guideline for the field of optical redox imaging, i.e., studies of mitochondrial metabolism and function based on intrinsic NADH and FAD fluorescence in cells, animal models, and human samples. The review by Talati and Tachtsitis^3^ focuses on hardware developments and future needs for broadband near-infrared spectroscopy (bNIRS) measurements that have facilitated *in vivo* measurement of cytochrome c oxidase oxidation. Lastly, Composto et al.^4^ review the latest technological and clinical advancements of hyperspectral imaging for guiding tumor resection during surgery.

Among the eleven research papers, six focus on novel device development and their applications for learning basic science in cell/animal disease models. Topics range from demonstration of a wireless miniaturized device for longitudinal monitoring of brain hemodynamic activities in free-moving mice by Bisht et al.^5^ to portable multi-parametric microscopy for noninvasive metabolic and vascular *in vivo* imaging of orthotopic tongue cancer models by Soumik Saha et al.^6^ Ochoa et al.^7^ report a new oversampling strategy utilizing the delayed fluorescence of protoporphyrin IX to enhance signal intensity in porphyrin-based hypoxia imaging. Benavides et al.^8^ demonstrate a widefield frequency-domain fluorescence lifetime imaging (WF-FLIM) system for label-free imaging of NADH and FAD in cancerous and noncancerous mammary tissue in mice. Hoang et al.^9^ investigate the effects of excess L-methionine on cellular metabolism and reactive oxygen species (ROS) accumulation in cell models of amyotrophic lateral sclerosis (ALS) using stimulated Raman scattering and optical redox imaging techniques; that work is featured on the issue cover. Ding et al.^10^ advance implantable biosensor technologies by integrating immunoassays with X-ray excited luminescence imaging for through-tissue protein detection.

Three papers are geared towards optical monitoring of human subjects and patients. Forcione et al.^11^ present a prospective study of healthy volunteers using a diffuse optical tomography system suitable for monitoring traumatic brain injury in the intensive care unit. Einalou et al.^12^ demonstrate the feasibility of noninvasive continuous blood pressure estimation using NIRS photoplethysmography and machine learning in patients undergoing carotid endarterectomy. Acharya et al.^13^ perform a pilot broadband near-infrared spectroscopy (bNIRS) study of hemodynamic and metabolic signals in patients with Alzheimer’s disease and report correlations between bNIRS and cognitive markers.

Lastly, two papers investigate the potential of NIR photo-biomodulation (PBM) as an intervention strategy for the central nervous system. A robust computational analysis of a rat model by Chen et al.^14^ provides insights to inform optimization of PBM parameters for treating spinal-cord injury, and the effects of NIR-induced transcranial PBM on visual functional changes were reported by Trofimov et al.^15^ in nearly 100 young, healthy volunteers.

In total, this special issue covers diverse contributions spanning physiological problems from cancer to brain function and injury. Some papers focus on responses in deep tissues in human subjects while other papers focus on the responses of cell models and preclinical small animal models. Finally, the research published in this special issue describes development of new devices and technologies for disease applications, and thus explores new biomedical science with state-of-the-art optical imaging/spectroscopy tools.
